# Effects of Soil Quality on the Microbial Community Structure of Poorly Evolved Mediterranean Soils

**DOI:** 10.3390/toxics10010014

**Published:** 2022-01-03

**Authors:** Antonio Camacho, César Mora, Antonio Picazo, Carlos Rochera, Alba Camacho-Santamans, Daniel Morant, Luis Roca-Pérez, José Joaquín Ramos-Miras, José A. Rodríguez-Martín, Rafael Boluda

**Affiliations:** 1 Cavanilles Institute for Biodiversity and Evolutionary, Universitat de València, E-46980 Paterna, València, Spain; cemoi@alumni.uv.es (C.M.); antonio.picazo-mozo@uv.es (A.P.); carlos.rochera@uv.es (C.R.); alcasan6@alumni.uv.es (A.C.-S.); daniel.morant@uv.es (D.M.); 2 Departament de Biologia Vegetal, Facultat de Farmàcia, Universitat de València, E-46100 Burjassot, València, Spain; luis.roca@uv.es (L.R.-P.); boluda@uv.es (R.B.); 3 Departamento de Didáctica de las Ciencias Sociales y Experimentales, Universidad de Córdoba, 14071 Córdoba, Spain; jjramos@uco.es; 4 Department of Environment, Instituto Nacional de Investigación y Tecnología Agraria y Alimentaria (INIA), E-28040 Madrid, Spain; rmartin@inia.es

**Keywords:** soil quality, soil microbiota, microbial diversity and evenness, bacteria, archaea, ectomycorrhizal Fungi, ecological networks

## Abstract

Physical and chemical alterations may affect the microbiota of soils as much as the specific presence of toxic pollutants. The relationship between the microbial diversity patterns and the soil quality in a Mediterranean context is studied here to test the hypothesis that soil microbiota is strongly affected by the level of anthropogenic soil alteration. Our aim has been to determine the potential effect of organic matter loss and associated changes in soil microbiota of poorly evolved Mediterranean soils (*Leptosols* and *Regosols*) suffering anthropogenic stress (i.e., cropping and deforestation). The studied soils correspond to nine different sites which differed in some features, such as the parent material, vegetation cover, or soil use and types. A methodological approach has been used that combines the classical physical and chemical study of soils with molecular characterization of the microbial assemblages using specific primers for Bacteria, Archaea and ectomycorrhizal Fungi. In agreement with previous studies within the region, physical, chemical and biological characteristics of soils varied notably depending on these factors. Microbial biomass, soil organic matter, and moisture, decreased in soils as deforestation increased, even in those partially degraded to substitution shrubland. Major differences were observed in the microbial community structure between the *mollic* and *rendzic Leptosols* found in forest soils, and the *skeletic* and *dolomitic* *Leptosols* in substitute shrublands, as well as with the *skeletic* and *dolomitic* *Leptosols* and *calcaric Regosols* in dry croplands. Forest soils displayed a higher microbial richness (OTU’s number) and biomass, as well as more stable and connected ecological networks. Here, we point out how human activities such as agriculture and other effects of deforestation led to changes in soil properties, thus affecting its quality driving changes in their microbial diversity and biomass patterns. Our findings demonstrate the potential risk that the replacement of forest areas may have in the conservation of the soil’s microbiota pool, both active and passive, which are basic for the maintenance of biogeochemical processes.

## 1. Introduction

It is well-established that the biodiversity patterns in terrestrial ecosystems are affected by anthropogenic activity. Although it is not always easy to define its consequences, this affects the way ecosystems work [1]. For instance, a reduction in richness also means the biodiversity’s diminished capacity to respond to destabilising elements as its resilience also decays [2,3]. Certainly, the presence of toxic pollutants is one of the most studied factors causing the decrease in the taxonomic and functional microbial diversity of soils [4,5]. Several authors [6,7,8] have reported that the presence of toxicants can have different origin, with the use of agrochemicals (fertilizers and pesticides) in agriculture being one of the main sources. On the other hand, forest fires can alter soil properties and the structure of microbial communities [9]. For instance, it has recently been reported that the combined effect of fire and the presence of chromium affects soil microbial communities [10], whereas toxics compounds such as dioxines or furanes are released into the environment by forest fires [11,12]. These substances can also alter the metabolism of soil microbiota [13]. However, physical alterations of soil and the changes in its chemical properties, though less studied from the point of view of its influence in soil microbiota, are critical as microbes play a key role by driving biogeochemical cycles in soils, thus influencing, for instance, soil fertility and, though indirectly, avoiding soil losses by keeping soil structure.

Soil quality is a concept that reflects either the general environmental status or acts as a model of present and future soil’s functional capacity [14]. Soil quality indicators include a wide range of biological processes and functions, which occur in situ, thus changes in soil quality must be reflected in the biological activities [15,16]. Based on this assumption, sound studies of the exogenous factors that cause negative alterations of soil quality are of interest. As an example, the European Union actively has established policies to protect and preserve soil quality and to maintain its biodiversity [17]. One way of dealing with such problems is to assess the spatial and temporal variation patterns in terms of species richness and the relation they have with certain functional traits [18]. The microbial biomass in soils accounts for most biological activity (60–80%) in the pedosphere, thus microbes are closely connected to the nutrient cycles and determine ecosystem stability and resilience [19]. It has long been argued that the soil type and its management might notably determine the microbial community structure in agricultural systems [20,21]. Moreover, biological variables are most valuable as early, sensitive indicators of changes in soil properties induced by different soil quality status. Generally, managed soils show, with some exceptions, a degraded vegetation cover associated with a decrease in soil organic matter and nitrogen content, respiration rates, microbial biomass C and enzymatic activities [15]. In the last two decades, techniques based on DNA extraction from soils and its selective amplification by PCR have been used to study the microbial communities of edaphic systems and their biodiversity [22,23,24,25]. The use of specific gene sequencing for microbial diversity studies is common in many studies. There are different techniques to approach this analysis, such as massive sequencing or analysis by denaturing gradient gel electrophoresis (DGGE). The analysis by DGGE allows to discriminate, through electrophoresis using polyacrylamide gels, differences of one nucleotide between amplicons, which is very useful in diversity studies, so this is still a useful technique to explore community patterns when multiple microbial groups are considered, as in our study [26]. Many studies into microbial soil diversity focus on a single taxonomic kingdom, either Bacteria, Archaea or Fungi [24,27,28], all of which may play different ecological roles. It is much less common, however, to find studies where a more integrative analysis, as we performed here, has been carried out: Here, the diversity patterns of all these microbial groups have been jointly studied by comparing them in soils showing different production potentials along a degradation gradient.

In this work we use the definition of soil quality as the ability of a specific soil type to operate, within the limits of natural and managed ecosystems, in sustaining the plant and animal productivity, in the maintenance or improvement of the environmental quality, and in support to human health [14,29]. Soil degradation is the decline in soil quality caused by its improper use, usually for human activities. In these conceptual aspects, the loss of vegetation due to abusive logging or fires induces soil organic matter loss, soil structure degradation, and negative changes in the chemical and biological properties, with a loss of soil functions and quality.

Soil degradation problems in the Mediterranean part of Spain have been widely reported, especially those related to desertification processes [30], soil use [31], and the presence of chemical pollutants [32,33,34,35,36]. Nevertheless, studies on the effects caused by anthropogenic edaphic alterations by changes in the vegetation cover on soil microbial activity are much scarcer [37]. In fact, though it has been addressed in other geographic areas [38], no study so far has covered the interplay of the soil degradation and anthropogenic disturbances and the richness of Mediterranean soils microbiota.

Therefore, the main objective of this work was to study the effects of soil quality and its degradation on microbial diversity in different edaphic systems in the chorological Ibero-Levantine area. In particular, the potential effect of organic matter loss was considered in relation with deforestation or agricultural use. We hypothesize that the physical and chemical changes caused either by deforestation or agricultural use alter the soil microbial communities and activity. The studied soils are distributed in three types according to the vegetation cover: forested lands, deforested areas showing serial shrubland vegetation, and zones used for dry croplands. Based on the results obtained, the potential risks that forest loss may pose on the pool of soil microorganisms, both active and dormant, are studied, thus highlighting the need to preserve vegetation cover in an area with a very high desertification risk, such as the Iberian Mediterranean region.

## 2. Materials and Methods

### 2.1. Study Area and Sampling

The soil samples used for this study originate from La Plana de Requena-Utiel, an inland region in the Valencian Community, located in Eastern Spain. Using a former pedological study [39], which included information about soil formation factors and soils distribution, areas with poorly evolved soils were selected showing three vegetation cover stages in the soil: natural (undisturbed forest), degraded shrubland, and agricultural edaphic systems, with different soil subunits within the two reference soil groups (Leptosol and Regosols) [40] whose organic matter content substantially varies due to the impact of human activities. Soil formation factors were identified and classified (Table 1). It is important to note that the areas of degraded shrubland (code C2, D2, and T2) are a consequence of the great fire that occurred in 1982 in this area. On the other hand, the areas dedicated to cultivation (code C3, D3 and T3) have been exploited for more than 60 years. The vegetation of the forest areas corresponds to holm oakwood of the lower supramediterranean belt belonging to the Bupleuro rigidi Quercetum rotundifoliae quercetosum rotundifoliae series; while in the case of the mesomediterranean belt the series corresponds to a mesomediterranean holm oak woodland with continental influence Bupleuro rigidi Quercetum rotundifoliae cocciferetum [39].

Samples were obtained from nine sites where geological features, vegetation, soils and their uses were identified. The sampled soils are representative of mountain ranges and calcareous valleys within the bioclimatic supra- and meso-mediterranean areas of the Iberian Peninsula. Sampled soils are coded according to its parent material (T: Marl and limestone; C: Limestone; D: Dolomites) and soil use (1: Forest; 2: Shrubland; 3: Crops) in such a way that, for example, C1 corresponds to a soil sample collected in a forest on limestone, which are poorly developed soils. Sampled soils correspond to the four following [40] FAO taxonomic soil subunits (2014): *mollic Leptosols,* on limestone (C1) and dolomites (D1) from the Cretaceous; *rendzic Leptosols,* on Tertiary limestone (T1); *skeletic Leptosols,* on Cretaceous limestone (C2, C3) and *dolomitic Leptosols* on dolomites (D2, D3); and *calcaric Regosols* on Tertiary calcareous detritic deposits (T2, T3) (Table 1). Within the nine studied sites, three had natural forest vegetation (sites 1). Two of them (C1 and D1) were closed formations, with holm (evergreen) oaks and mountain pine shrublands, mixed with more open formations with holm oaks belonging to the basophil supramediterranean vegetation series of holm oak with a continental influence: *Bupleuro rigidi-Quercetum rotundifoliae-quercetosum rotundifoliae.* The third forested site (T1) had a basophilous mesomediterranean vegetation series of the holm oak *Bupleuro rigidi-Quercetum rotundifoliae cocciferetum* (T1). The other three sites displayed a potential vegetation degraded to substitution shrubland (sites 2), made up of thyme swards, where *Erinacea anthyllis* and *Salvia lavandulifolia* were abundant (C2 and D2), plus serial shrublands with Kermes oaks (*Quercus coccifera*), and thyme swards where *Rhamnus lycioides* was present (T2). Finally, there were three sites located in croplands (sites 3), with cereals (C3 and D3), vineyards and almonds crops (T3).

Four zigzag systematic samplings were performed at an equal distance of 50 m at each study site. For each sampling plot, four subsamples were obtained, combined and homogenised to give a composite sample of approximately 5 kg. At each site, the A-horizon was sampled from 0 to 15 cm by digging a trench of about 30 × 30 × 15 cm in size, taking a representative sample from this soil volume. Additionally, in forested areas, the organic horizon (O) was also sampled by taking forest litter, considered as being the superficial, heterogeneous and low decomposed layer of the O horizon (O-L) (C1L, D1L and T1L samples) and humus, considered as the deep, homogeneous, dark coloured and high decomposed layer of the O horizon (O-H) (C1H, D1H and T1H samples). From each fresh sample, one part was sieved in the field to fill a container of 200 cm^3^ in order to determine the carbon biomass. Furthermore, four cylinders (5 cm in diameter and 7 cm length) were taken from each plot to determine bulk density. These containers and cylinders were placed inside a portable ice-cooler to be later stored in a chamber at 4 °C until analysed. In order to determine microbial diversity using molecular biology techniques, 100 g of soil were taken from the composite sample at every site, then they were stored frozen (−20 °C) until analysed. The rest of analyses were performed from the remaining composite sample.

### 2.2. Soil Characteristic Determinations

The parameters directly related to typology and soil quality were determined using standard methods. Samples underwent a pre-treatment, which was performed following the ISO 11464 procedure [41]. The pH was measured in a soil-water suspension 1:5 (*v*/*v*) [42]. Electrical conductivity (EC) was measured in an aqueous soil extract at a 1:5 ratio (*w*/*v*) [43] using a Crison conductivimeter. The equivalent calcium carbonate content was determined by gasometry with a Scheibler device [44]. To determine the bulk density, a known soil volume was extracted by means of a cylinder and was oven-dried, then weighed to establish the weight/volume ratio [45]. In order to determine water-holding capacity (WHC), samples were submitted to the pressure plate method (pF = 2.5) described by Richards [46]. Particle size was measured by following the Bouyoucos [47] densimeter method. Total N, C and S contents were determined by heating up to at least 900 °C in the presence of oxygen using an EA 1110 (CE Instruments) CHNS elemental analyser [48,49,50]. To determine the cationic exchange capacity (CEC) and exchangeable Na, K, Mg and Ca content, the soil saturation method with barium chloride was used, with the subsequent displacement of Ba^2+^ by a known excess of magnesium sulphate [51]. Soil organic matter (SOM) was determined by oxidation with potassium dichromate and sulphuric acid, with subsequent titration of the excess of dichromate with ammonium ferrous sulphate [52]. The microbial carbon biomass analysis was done according to the substrate-induced respiration method, based on the fact that during the first hours after adding an excessive amount of glucose to a sample, soil microorganism’s respiration was limited only by the abundance of their microbiota. Then, the maximum initial respiratory response would be in proportion to the microbial carbon biomass [53]. Biologically available phosphorus was determined by extraction with 0.5 N NaHCO_3_ at pH 8.5 [54]. All analyses were done in triplicate.

### 2.3. Molecular Methods

For DNA extraction and purification, 250 mg of each sample were processed using a commercially available kit (Powersoil DNA Isolation Kit, MoBio Laboratories Inc., Carlsbad, CA, USA) following manufacturer’s protocol. Extracted DNA was quantified by fluorescence with SYBR-Green-I to check the efficiency of the extraction method by comparing the result with the microbial biomass analysis. The study was done by PCR amplification of the V3-V4 hypervariable region of the 16S rRNA gene of Bacteria and Archaea, and the ITS18S-region for ectomycorrhizal Fungi. The mix per reaction (20 µL) consisted in: 2 µL of 10X Tris HCl buffer, 0.6 µL of Cl_2_Mg 50 mM, 0.25 µL of dNTPs 10 mM, 0.25 µL of each primer, 1 µL of BSA, 2 µL of polymerase, 7.65 µL of Milli-Q ultrapure water and 6 µL of the DNA sample. For Bacteria the following primers were used: 341F-GC (5′-CGC CCG CCG CGC GCG GCG GGC GGG GCG GGG GCA CGG GGG GCC TAC GGG AGG CAG CAG-3′) and 907R (5′-CCG TCA ATT CCT TTG AGT TT-3′). The program setting was 5 min at 94 °C, 1 min at 80 °C, 35 cycles at 94 °C 1 min, 45 °C 1 min and 72 °C 1 min, and a final elongation step at 72 °C for 30 min [55]. For Archaea, primers used were: 344F-GC (5′-CGC CCG CCG CGC CCC GCG CCC GTC CCG CCG CCC CCG CCC CAC GGG GYG CAG CAG GCG CGA-3′) and 915R (5′-GTG CTC CCC CGC CAA TTC CT-3′). In this case, a PCR touchdown was performed: 94 °C 5 min, 20 cycles at 94 °C 1 min, 71 °C (−0.5 °C per cycle) 1 min and 72 °C 3 min, followed by 15 cycles at 94 °C 1 min, 61 °C 1 min and 72 °C 3 min, with a final elongation step at 72 °C for 10 min [56]. For ectomycorrhizal Fungi the following primers were used: ITS4B-GC (5′-CGC CCG CCG CGC CCC GCG CCC GGC CCG CCG CGC CCG GCC CAG GAG ACT TGT ACA CGG TCC AG-3′) and ITS1F (5′-CTT GGT CAT TTA GAG GAA GTA A-3′). The program setting was 94 °C 3 min, followed by 40 cycles at 94 °C 1 min, 48 °C 1 min and 72 °C 1 min, and a final elongation step at 72 °C 10 min [57]. All the amplifications were carried out in an Eppendorf Mastercycler Personal thermocycler.

Once PCR was completed, amplification was checked by agarose gel electrophoresis (1% agarose, 120 V, 1 h) by taking an aliquot of the PCR product and placing a marker in the gel to verify the size of the amplified bands. 16S and ITS rDNA-DGGEs were performed by using the CBS System (CBS Scientific Company). A total of 500 ng of the PCR product was loaded on a 7% polyacrylamide gel (Acrylamide/Bisacrylamide 37.5:1) containing a denaturant gradient of 30–70% for 16S rRNA (Bacteria and Archaea) and of 20–60% for 18S (fungal ITS) made by urea and formamide. Gels were electrophoresed at 60 °C at a constant voltage (100 V) for 17 h and were stained for 1 h using SYBR-Green I. Bands were recorded to digital images by UV light gel transillumination. Scanned gels were analysed with the Quantity One software package (Bio-Rad). The detected peaks were manually adjusted to eliminate unresolved peaks arising from the background and the band intensities were calculated by measuring the peaks of the bands in the histogram as described by Chan et al. [58]. The number and intensity of bands (each band define an Operational Taxonomy Unit -OTU-) were used as parameters for further analyses of diversity.

### 2.4. Data Analyses

For the environmental data (Euclidean distance), as well as for the DGGE profiles for the different microbial groups, Bacteria, Archaea, and ectomycorrhizal Fungi, (Bray Curtis similarity), a cluster analysis was performed. Dendrograms for DGGE profiles were built-up using the band’s relative intensity matrix. SIMPROF test for statistical significance of dendrogram branch was performed with software Primer 6.0 (sig. level: 5%, and number of permutations: mean: 1000, simulations: 999).

N1 exponential Shannon index was used to control the variability associated with rare taxa by differentially weighting them [59]. Due to the generality and flexibility in controlling the effects of rare taxa in microbial communities, Hill number was used as being an excellent tool for microbial diversity studies [60]. Evenness index was calculated as Pielou’s eveness J’ [61]. All diversity calculations were carried out with Primer 6.0 software.

In order to analyse the influence of environmental gradients on the fingerprint profile of communities, a cluster analysis (HeatMap), as well as a Distance-based redundancy analysis (dbRDA), were performed based on DGGE band intensity. This was performed based on the Bray–Curtis dissimilarity [62]. HeatMap workflows were carried out using “pheatmap” package (version 1.0.12) in the R programming environment [63] to describe community dissimilarity in unconstrained space. Distance-based redundancy analysis (dbRDA), which describes the microbial community structure ordinations in the environmentally constrained space, was performed using Primer 6.0 software.

ANOVAs, with post hoc tests (Scheffe ≤ 0.05), were also performed for the comparison of environmental variables (Sand, Silt, Clay, Density, Water Holding Capacity, Electrical Conductivity, pH, CaCO_3_, Carbon %, Sulphur %, Cation Exchange Capacity, Exchangeable calcium, Exchangeable magnesium, Exchangeable potassium, Exchangeable sodium, Phosphorus pentoxide, Nitrogen %, Soil Organic Matter, Microbial Biomass) among the different soil uses. With the same variables, an Unweighted Pair Group Method with Arithmetic Mean (UPGMA) cluster analysis, with a standardised euclidean distance, was also performed.

### 2.5. Community Interaction Networks

Networks were constructed on the basis of the intensity band of DGGE profile using CoNet software [64]. We constructed an interaction network based in the combination, for each edge, of Bray-Curtis distance and Pearson correlation between all DGGE bands. Interactions network between DGGE bands were obtained with a Bray-Curtis threshold of 0.4, and threshold Pearson correlation 0.9 was assigned to a specific factor if its relative band intensity in that factor was higher than 90% of the sum of the intensity for all factors. The final network was visualized with the yfiles organic layout from Cytoscape network visualizing software [65].

## 3. Results

### 3.1. Soil Use and Soil Properties

The studied soils are poorly evolved. The main mineral horizons are A and C, as well as a conspicuous organic horizon (O) in forest soils which includes mainly forest litter at different stages of decomposition (L, H). Another well-defined layer was R (in limestone and dolomite rocks). Overall, the soil profiles showed the following horizon sequences: O-Ah-R for *mollic* and *rendzic Leptosols*, and AC to *calcaric Regosols* (Table 1).

The main properties of the studied soils are presented in Table 2, and the post hoc analyses of these data to show the statistically significant differences among the studied soils are given in Table 3. Regarding physical properties, the water holding capacity (WHC) sharply dropped in parallel with increased soil degradation with values that were on average 14% lower in agricultural soils and 10% lower in shrubland compared to forested areas, while soil bulk density displayed the opposite behaviour with almost triple values in agricultural soils compared to forests. Soil texture ranged from silty-clay-loam to silty-clay.

**Figure 1:**
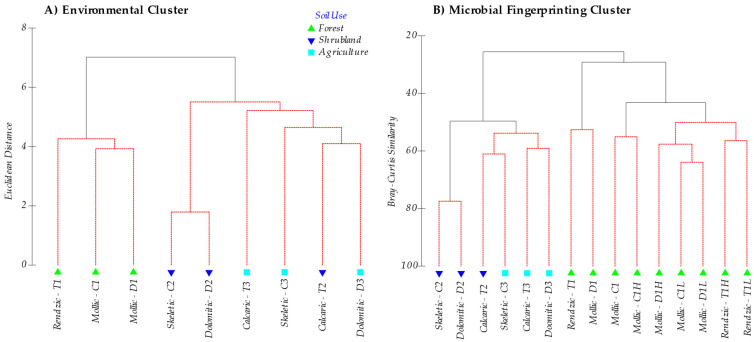
(**A**) Cluster analysis based on Euclidean distance of all environmental data (Table 2). (**B**) Cluster analysis derived from DGGE (fingerprinting) analysis of the microbial community based on the Bray Curtis similarity with the results of all the DGGE profiles for the different microbial communities analysed (Bacteria, Archaea and ectomycorrhizal Fungi). Note that, for the latter, the sub-horizons of forest soil are differentiated. Soils are labelled as parent material (T: Marl and limestone; C: Limestone; D: Dolomites), soil use (1: Forest; 2: Shrubland; 3: Crops) and O-horizon for the forest soils (L: Litter; H: Humus). Red lines in cluster are SIMPROF test for statistical significance (sig. level 5%), and number of permutations (Mean: 1000, simulations: 999).

In general, soils ranged from a slightly basic pH (7.35–7.70) in forest areas to a basic pH (8.34–8.45) in agricultural soils, and showed similar EC properties (soils without salinity problems). Carbonates, CEC, exchangeable Ca and Mg, SOM, N and microbial C-biomass differed considerably between the different serial vegetation stages, vs. levels of degradation and type of soils. Soil organic matter content was generally high, except for those soils with an anthropic-Ap horizon (cultivated soils). When analyzing the integrated sample, although no statistically significant differences were found in the post hoc test (Scheffe) for Na, K and P (available P_2_O_5_) contents, higher P_2_O_5_ values were found in forest and agricultural soils (mean value for these groups is 2.03 mg 100g^−1^, Table 3) when compared to shrublands (0.96 mg 100g^−1^), which relates to the use of mineral fertilizers in crops and natural fertilization in forests. SOM and N contents were much higher (16.66% and 0.68%, on average, respectively) in forest soils compared to shrubland (6.37% and 0.356%, respectively) and crop soils (2.37% and 0.19%, respectively). Cluster analysis (Figure 1A) separated forest soils (1) from shrublands (2) and those (mostly) from crop soils (3).

### 3.2. Soil Microbial Biomass and Diversity (Analysis of Soil Microbial Communities)

Microbial biomass (Table 2) can be indirectly represented by induced respiration as it shows a significant positive correlation (R^2^ = 0.97; *p* < 0.001) with the amount of extracted DNA as a surrogate of biomass, implying that they are basically proportional to the microbial biomass in the samples, and are not mainly a result of a differential metabolic status among the communities of the different studied soils. The results reveal statistically significant differences (alpha = 0.05, Table 3) related to the soil use. Higher values where consistently seen for *mollic* and *rendzic Leptosols* from forested areas, medium values for the *Leptosols* (*skeletic* and *dolomitic*) of shrubland, and the lowest values for the *Leptosols* (*skeletic* and *dolomitic*) with crops and *calcaric Regosols*.

In all the soils studied, DNA amplification was obtained for all three groups of the analysed microorganisms (Bacteria, Archaea and ectomycorrhizal Fungi). Figure 1B shows the Cluster analysis of the bands pattern (OTU’s, Operational taxonomic Units) obtained for the microbial groups by DGGE in all the analysed soils, making up the microbial population structure. The cluster analysis clearly grouped on one side forest soil samples (sites 1) (including O-L and O-H horizon samples) separated from shrubland soil samples (sites 2), which were also disaggregated from cropland soils (sites 3).

**Figure 2:**
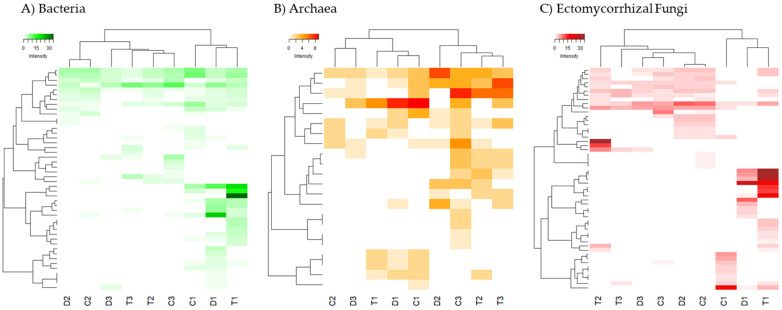
Heatmap of two-way cluster analysis performed on the fingerprinting profiles of the DGGE analysis for (**A**) Bacteria. (**B**) Archaea and (**C**) ectomycorrhizal Fungi. Both fingerprinting profiles and samples were clustered using Bray-Curtis dissimilarities. The colour intensity in the cluster dendrogram corresponds to the abundance of normalized intensity of the DGGE bands.

A heatmap analysis (Figure 2) was performed separately for the three groups of microorganisms studied (Bacteria, Archaea and ectomycorrhizal Fungi). For Bacteria and ectomycorrhizal Fungi there were specific taxonomic units that were characteristic of the forest floor, even with specific OTUs for the three types of forest soils studied (T1, C1, D1). Instead, no such differentiated clades were observed for shrubland and agricultural soils. For Archaea, although the forest soils are still grouped together, these differences are much more blurred.

**Figure 3:**
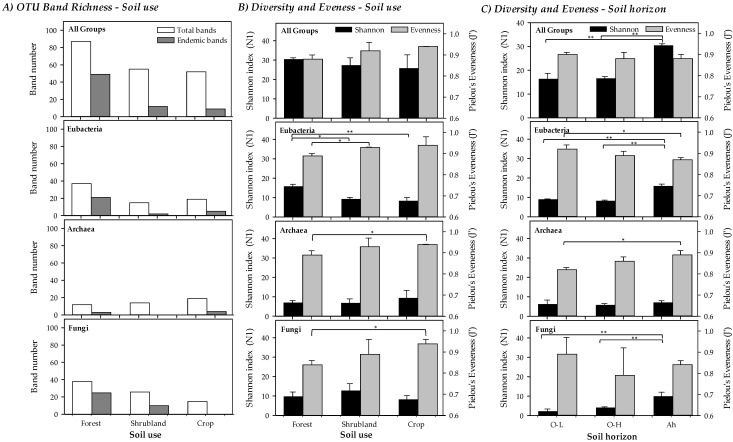
(**A**) Band richness, (**B**) Shannon Diversity (N1) and Pielou’s Evenness (J’) indices measuring microbial diversity (Total, Bacteria, Archaea and ectomycorrhizal Fungi) for the different soil uses (forest, shrubland and crops) in the A-horizon. Statistically significant differences in Shannon Diversity (N1) and Pielou’s Evenness (J’) among soil use types are marked (* *p* < 0.05; ** *p* < 0.01). (**C**) Shannon index (N1) and Pielou’s Evenness (J’) at different soil horizons in forest soils (Total, Bacteria, Archaea and ectomycorrhizal Fungi). Statistically significant differences among soil use types are marked (* *p* < 0.05; ** *p* < 0.01).

The band richness and number of endemic bands (considered as bands that appear only in a soil use, forest, shrubland or crops) of the A-horizon are shown in Figure 3A. A total of 87 different bands (OTUs) were obtained in forest soils, 55 in shrubland and 52 for crops. Of these, a high number (49) were endemic for forest while only 12 and 9 were endemic for shrubland and crops, respectively. Particularly, major differences were found for Bacteria and ectomycorrhizal Fungi, with a clearly higher number of bands of both groups in forested areas. For Bacteria 37 (21 endemic) different bands were found in forest soils, 15 (2 endemic) in shrubland and 19 (5 endemic) in crops. For ectomycorrhizal Fungi 37 (25 endemic) bands were found for forest soils, 26 (10 endemic) in shrubland and 15 (none endemic) in crops. For Archaea lower differences were found, with 13 (3 endemic) bands in forest soils, 14 (none endemic) in shrubland and 18 (4 endemic) in crops.

The Shannon Diversity and Evenness indices obtained from the DGGE profiles for all soil uses (Figure 3B) and soil horizons (just for forested soils, Figure 3C) are given for all groups as well as separately per taxonomic group. It should be highlighted that the Evenness results for microbial communities are usually very high if compared to other ecological studies where the Shannon index is calculated counting individuals (e.g., macroorganisms). Values of the Shannon diversity index were slightly higher, though this was not statistically significant, in less degraded soils, in part due to the low number of cases compared as well as by the compensatory effect produced by the Evenness, as band intensities were more similar in more altered soils. Statistically significant differences among the three soil uses were only found for Bacteria, being the more diverse forest soils (Shannon index) than shrubland and crops for this group. Evenness, however, presented a pattern with an increasing trend with increased soil degradation, regardless it was jointly considered for all groups or separately for each one, showing statistically significant lower values between forest and crop soils for Archaea and ectomycorrhizal Fungi. Statistically significant differences, though with different patterns for the different taxonomic groups studied, were found when soil horizons of forested areas were compared (Figure 3C), as described in Section 3.4.

### 3.3. Relationship between Microbial Diversity and Soil Characteristics

**Figure 4:**
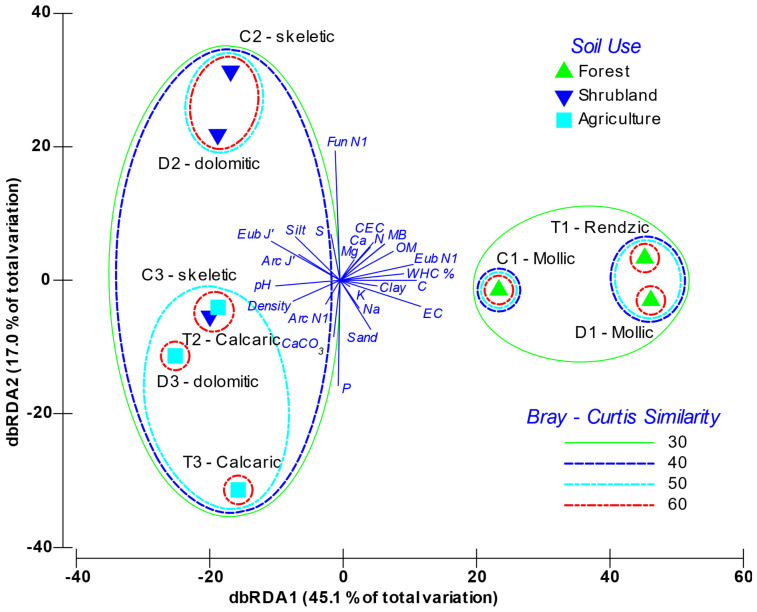
Distance-based redundancy analysis (db-RDA) triplot between environmental variables and the fingerprinting profiles of the A-horizon soil samples. Soils are labelled as parent material (T: Marl and limestone; C: Limestone; D: Dolomites) and soil use (1: Forest; 2: Shrubland; 3: Crops). EC: Electrical Conductivity; WHC: Water Holding Capacity; CEC: Cation Exchange Capacity; Ca: Exchangeable calcium, Mg: Exchangeable magnesium, Na: Exchangeable sodium, K: Exchangeable potassium, SOM: Soil Organic Matter; MB: Microbial Biomass; E: Evenness; H’: Shannon Index; Arc: Archaea; Eub: Bacteria; Fun: Ectomycorrhizal Fungi.

The dbRDA analysis (Figure 4) graphically depicts the distribution of the variables and the soils’ factor scores within the two main principal components axes. The first component accounted for 45.1% of variability, while the second one explained 17.0%. The analysis clearly grouped soils according to use, especially within the first component axis; whereas there was a second level of discrimination based on soil’s typology. The first axis of the dbRDA was positively related with the soil’s organic richness, where the more significant variables were microbial biomass, diversity of Bacteria (Eub H’), SOM, N and C. On this axis, other variables associated with the soil chemical characteristics were also weighted, such as conductivity, CEC, exchangeable Ca and Mg, and the water holding capacity (WHC). Non-altered forest soils (*mollic* and *rendzic Leptosols*) were associated to the positive side of axis 1. The most altered soils *(skeletic* and *dolomitic Leptosols* with crops and *calcaric Regosols*) were distributed in the negative part of this axis, and were strongly related with pH, density and CaCO_3_, and, for the microorganisms, more slightly related to the Archaea diversity (Arc H’) and the Evenness for Bacteria, Archaea and ectomycorrhizal Fungi. On the other hand, axis 2 was mainly determined by P opposed to S content, and by soil particle-size distribution. With regard to the biological parameters, this component was strongly influenced by fungal and archaeal diversity with opposite weighting. Figure S1 shows how, independently of including physical-chemical or biological variables, or both, the sample clustering was very similar, which demonstrates that soil features and soil microbiota interacted. In all cases, the forest soils grouped, as did the *skeletic* and *dolomitic Leptosols* both with shrubland, and the *Leptosols* with crops mixed with the *calcaric Regosols*. Sample T2 (Shrubland calcaric) grouped with the crop samples, as the calcaric component has a relevant specific weighting explaining the microbial variability.

### 3.4. Microbial Diversity of the Different Forest Soil Organic Horizons

In addition to the Ah-horizon in forest soils, we also analysed the diversity of the O-horizon: humus (O-H) and forest litter (O-L) layers. In the O-L 40 different bands (OTUs) 15 Bacteria, 18 Archaea and 7 ectomycorrhizal Fungi were found in total. In the case of O-H layer a markedly different distribution was found, with 13 bacterial bands, 10 for Archaea and 17 for ectomycorrhizal Fungi.

The Shannon index (N1, Hill number) and Evenness of the three different forest soil horizons are compared in the Figure 3C. Overall, Shannon diversity of both O-horizons was similar (16.42 for O-H and 16.59 for O-L) but significantly lower than in the Ah-horizon (30.46). In particular, the diversity in the Ah-horizon was always significantly higher except for Archaea. A similar trend than in band richness was found for diversity when O-H and O-L were compared, with similar diversity for Bacteria, with a higher diversity for Archaea in O-L and a higher diversity for ectomycorrhizal Fungi in the O-H layer.

Concerning Evenness, a clear pattern was not found. Overall, it was slightly higher in the O-L, but not significantly different. In particular, Evenness was higher in the O-L for Bacteria and ectomycorrhizal Fungi, but significantly lower for Archaea. In the case of O-H, a low Evenness for ectomycorrhizal Fungi combined with a high deviation value appeared, due to the presence of a very intense band in the *rendzic Leptosol*.

### 3.5. Microbial Network Analysis

**Figure 5:**
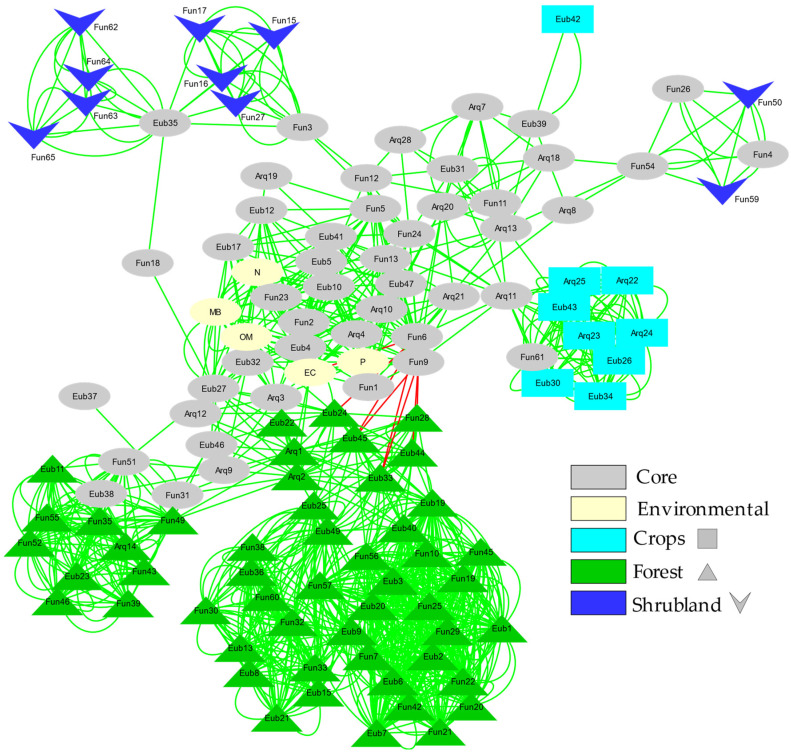
Bray Curtis and mutual exclusion Network of the fingerprinting profiles for Bacteria, Archaea and ectomycorrhizal Fungi. Each factor (Forest; Shrubland; Crops) presents its own code of colour, the nodes were assigned to a specific factor if the relative band intensity represents more than 90% in that factor. If the relative abundance was lower than 90% and the ZOTU was shared between others factors it was classified as a Core node.

Network analysis (Figure 5) of the relationships between the different taxonomic units detected by fingerprinting (both among them and with respect to the environmental variables studied) shows how the cosmopolitan (present in most soil types) and endemic OTUs of each soil type (forest, shrubland and agricultural) develop sub-networks around cosmopolitan OTUs (core).

Both the size and the number of connections of these sub-networks denote the stability and complexity of the interactions between the studied microorganisms (Bacteria, Archaea and ectomycorrhizal Fungi). The simplest and poorly connected networks are the shrubland and agricultural ones. The forest soil sub-network is the largest and most complex, consisting of two separate subunits connected to each other.

## 4. Discussion

The main objective of this study was to establish to what extent soil conditions altered by some human activities influence the patterns of microbial diversity of Mediterranean ecosystems, where the decrease in soil quality is understood as a loss of potential vegetation linked to a gradual decrease in SOM associated with human activity [66,67]. The results of Table 3 coincide with those reported by Boluda et al. [15,39], who found wide variations of soil properties due to changes in the soil formation factors, particularly vegetation, climate, and soil uses in this area, which relates soil biology with soil quality. The results obtained here indicate that human impacts, such as deforestation (e.g., forest fire) and agricultural practices (the use of fertilizers and pesticides), lead to negative changes in soil attributes and shifts in the microbial diversity. This agrees with previous results reported by several authors, both for the effects of fires [68,69] as well as for the effect of chemicals used in agriculture [70,71]. Because of these impacts, changes in the morphological characteristics of the soil profile are brought about: the O-horizon disappeared [15,72,73], the *mollic* Ah-horizon in *mollic Leptosols* changed into an Ah-ochric diagnostic horizon under shrubland communities (*Leptosols)*, and into an anthropic diagnostic horizon given agricultural practices [74,75]. These changes also cause variations in some soil properties; for instance, decreased WHC, SOM, CEC and nutrients, and increased bulk density, soil pH and calcium carbonate content (Table 3). A lower soil pH in relation with increased SOM is a well-known phenomenon, and could also affect leaching processes [76,77]. This would facilitate leaching of Ca and Mg from exchangeable complexes, while the loss of SOM contributes to the structure instability. Thus, it is evident that the changes in soil quality caused by different land uses must first be quantified to establish the most sustainable uses and management causing lower disturbance to soils. Given that soil quality depends on its physical, chemical, biological and biochemical properties [15,78], changes in these properties must be considered when assessing changes in soil biodiversity.

Our study demonstrates that the changes in the soil quality are caused by land use-related human activities (Table 1), unveil by the loss of the organic horizon (O) in the soil profile. Biochemical decomposition and incorporation of the fresh organic matter present in the O-horizon is performed by soil microorganisms. This material is modified through biodegradation and humification by a wide variety of biological and biochemical mechanisms [79], being favoured by a higher microbial diversity as holding more potential metabolic capacities. Clearly, human impacts contribute to decrease SOM and N contents, as well as to a drop in microbial biomass-C and richness, as soil quality decreases (Table 3). Accordingly, the soil quality sequence is: *mollic Leptosols* > *rendzic Leptosols* > *other Leptosols* > *calcaric Regosols*. All these aspects are related with changes in soil microbiota, which was here detected by applying molecular techniques.

Making estimations of the microbial community abundance and structure with molecular techniques has some limitations [80], since some known biases may occur while amplifying target DNA fragments. In our case, special care was taken when establishing the number of minimum PCR cycles, as well as in relation to the need of amplifying all the samples in the same PCR analysis, also including using the same gel for DGGE and the same staining and photographic exposure of all the samples. Although this limits the number of samples being treated, it lowers the possible bias in the obtained results and enables a suitable comparison of the microbial diversity patterns among the studied samples accounting for all important microbial groups in soils (Bacteria, Archaea, and ectomycorrhizal Fungi). A high confidence arises from the fact that the correlation found between microbial biomass and the DNA amount extracted from each soil sample shows a significant Pearson’s correlation coefficient of 0.974. Such adjustment ensures that DNA extraction was nearly quantitative and in proportion to the microbial biomass, which on this aspect rules out a possible bias.

Classifying soils according to the band patterns obtained in the fingerprinting analysis (DGGE) shows a primary and major separation among the *mollic* and *rendzic Leptosols,* found in a potential forest setting, with the *skeletic* and *dolomitic Leptosols* in substitute shrublands, and the *skeletic* and *dolomitic Leptosols* and *calcaric Regosols* of dry croplands. What clearly comes out is that microbial composition differs at each soil quality level in accordance with a SOM, N and C biomass content gradient, irrespective of the soil type. The microbial composition of the humic layer and forest litter from the O-horizon is closely related with that of the A-horizon [81], suggesting that the succession of the microorganisms in the degradation process of both forest litter and humus continues during soil formation. Litter and humus from the O-horizon do not show a clearly distinct community composition, and they cluster mixed (Figure 1), which shows that humification is a continuous process with a parallel succession of microorganisms. Yet, although with more differences, the set of samples from the O-horizon (litter and humus) further cluster with the soil samples of unperturbed forest. Thus, loss of O-horizon in shrublands explains the change in its soil community composition in relation to the natural undisturbed forest, then, the microbial composition of shrublands soils is closer to that observed in the crop soils studied (Figure 1 and Figure 4). This is the first main consequence of the degradation process, that is, the strong change in the microbial population structure after deforestation and the loss of the O-horizon.

Changes in the microbial composition of the different studied soils may be reflected in diversity indices in several ways [82,83,84]. Diversity estimated by the Shannon Index (H’) combines evenness and the number of species. In general, we found a larger number of species (OTUs) when the level of soil quality was high. However, when we jointly checked all the microbial groups studied, we found no statistically significant differences in diversity (H’) among the various soils’ uses (Figure 2). Instead, there is a significant difference in Evenness, which increases with the level of degradation. Irrespective of the diversity pattern noted for each microbial group studied, the Evenness pattern is always similar, increasing with a trend to increase soil quality degradation. This could be partly related to the disturbance regime, as croplands are frequently altered by agricultural works and thus the ecological competitive scenario is not quite stable, which would lead to a more shared (although less rich) composition of the community. On the contrary, forest soils correspond to a nearly climax community where disturbance levels are reduced and dominance (in terms of abundance) of some taxa would drive towards reduced Evenness, even though richness is higher [23,24,82]. This seems to be confirmed by the higher stability of the forest soil sub-network, which, as shown by the network analysis (Figure 5), is the largest and most complex sub-network consisting of two separate subunits connected to each other. Additionally, this effect can also be enhanced by ectomycorrhizal Fungi, due to the fact that these microorganisms are linked with the roots of the aboveground vegetation [25]. The higher band intensities (and the lower Evenness) were found for ectomycorrhizal Fungi in forest and shrubland soils, probably due to the linkage of these fungal species with the aboveground dominant vegetation species inhabiting the zone. This also can explain the low diversity found for ectomycorrhizal Fungi in the O-L layer, due to the lack of roots in this horizon.

A sounder study was focused on the number of endemic bands. This showed that forest soils not only have more species, but also that these species are very different to those found in shrubland and crops, where we found a lot of coincident bands between samples of these two soils uses. This shows the extent to which soil degradation implies the loss of many unique species.

Less disturbed soils, rich in SOM, hold more stable populations with more different species (a total of 87 in *mollic* and *rendzic Leptosols*), particularly for Bacteria and ectomycorrhizal Fungi, although some species may become quite dominant. With the organic horizon lost, soils undergoing degradation present a lower number of species (a total of 55 OTUs in shrublands and 52 in cultivated soils), although the lower dominance balances Evenness.

Furthermore, the results obtained in the dbRDA, based on both the physical-chemical variables and the microbial community structure, reveal, in the main axis, which accounts for 45.1% of variability, a clear separation of the studied samples depending on soil type and soil quality level, as well as accordingly with the SOM gradient. Overall, there are not only the physical-chemical variables that determine this samples distribution, since the dendrogram, which groups the analysed soils according to biomass, diversity and evenness for the microbial community, shows a similar clustering to that observed with the physical-chemical variables (Figure 4). Thus, this clustering is similar regardless of whether it comes from the physical-chemical variables or the biological variables, which shows the interplay between soil features and alteration and the microbial community structure.

In summary, in our case, the soil quality decrease in this Mediterranean area can be understood first as a loss of SOM due to deforestation processes, and secondly as the result of exhaustion brought about through crop-growing action. Hence, we may conclude that loss of the organic horizon leads to a major change in the microbial populations of Bacteria and ectomycorrhizal Fungi, but to minor changes for Archaea diversity. These changes bring the population structure of shrubland soils (forest loss) close to that of cultivated soils. Yet, bearing in mind the physical-chemical variables and microbial diversity, it can clearly be observed that soils are grouped according to their quality, which, although no statistically significant differences in biodiversity (H’) are generally observed along the soil quality loss, shows that better quality soils (*mollic* and *rendzic Leptosols*) present less Evenness and a larger number of species (both common and endemic) than more disturbed soils (*skeletic* and *dolomitic Leptosols* and *calcaric Regosols* from shrubland and crop areas).

Future research resulting from this work would need a more detailed study of soil’s microbial composition in a degradation gradient [85]. This can be done, for example, by identifying the different species found in each soil and studying their ecological capacities and biogeochemical role, in order to assess the ecological consequences derived from the changes in the microbial communities linked to soil degradation through changes in use. This would help for the selection of land-use management practices [20] to improve the soil quality in Mediterranean areas, which are strongly affected by a desertification process that can be enhanced by climate change.

## Figures and Tables

**Table 1:** Summary of the studied soils showing their uses, soil formation factors, horizons, and taxonomy.

Sample Code	Soil Use	Bioclimatic Belt	Parent Material	Vegetation	Soil Horizon	Soil Type (FAO)
C1	Forest	Supra-Mediterranean	Limestone	Holm Oakwood	Mollic (Ah)	*mollic Leptosol*
C2	Shrubland	Supra-Mediterranean	Limestone	Shrubland	Ochric (Ah)	*skeletic Leptosol*
C3	Crops	Meso-Mediterranean	Limestone	Cereals	Anthropic (Ap)	*skeletic Leptosol*
D1	Forest	Supra-Mediterranean	Dolomites	Holm Oakwood	Mollic (Ah)	*mollic Leptosol*
D2	Shrubland	Supra-Mediterranean	Dolomites	Shrubland	Ochric (Ah)	*dolomitic Leptosol*
D3	Crops	Supra-Mediterranean	Dolomites	Cereals	Anthropic (Ap)	*dolomitic Leptosol*
T1	Forest	Meso-Mediterranean	Marl and limestone	Holm Oakwood	Mollic (Ah)	rendzic Leptosol (*)
T2	Shrubland	Meso-Mediterranean	Marl and limestone	Shrubland	Ochric (Ah)	*calcaric Regosol*
T3	Crops	Meso-Mediterranean	Marl and limestone	Almonds and vineyards	Anthropic (Ap)	*calcaric Regosol*

(*) Has a continuous rock stratum starting ≤ 25 cm depth.

**Table 2:** Physical, chemical and biological properties of the A horizon of the studied soils. Soils are labelled regarding the parent material (C: Limestone; D: Dolomites; T: Marl and limestone) and soil use (1: Forest; 2: Shrubland; 3: Crops). Data are shown as mean ± standard deviation. WHC: Water Holding Capacity; EC: Electrical Conductivity; CEC: Cation Exchange Capacity; ExCa, Exchangeable calcium, ExMg, Exchangeable magnesium, ExNa, Exchangeable sodium, ExK, Exchangeable potassium, SOM: Soil Organic Matter; MB: Microbial Biomass (in carbon).

	C1	C2	C3	D1	D2	D3	T1	T2	T3
Sand %	27 ± 7.16	17.75 ± 0.96	25.25 ± 2.06	24.75 ± 1.26	13.5 ± 3.51	19.75 ± 2.36	30.25 ± 2.36	39.25 ± 6.18	28.5 ± 0.58
Silt %	35.75 ± 2.36	41.5 ± 9.85	41.75 ± 3.77	32.5 ± 6.14	43.5 ± 5.26	42.25 ± 2.87	25.75 ± 6.65	25.25 ± 4.92	28.5 ± 3.51
Clay %	37.25 ± 5.56	40.75 ± 10.24	32.5 ± 1.29	43 ± 5.23	42.75 ± 3.10	37.75 ± 0.50	44 ± 4.69	35 ± 2.94	43 ± 3.56
Bulk density (g cm^−3^)	0.44 ± 0.03	0.81 ± 0.03	1.28 ± 0.11	0.47 ± 0.04	1.05 ± 0.05	1.23 ± 0.02	0.68 ± 0.06	0.94 ± 0.10	1.17 ± 0.16
WHC %	21.85 ± 1.43	12.63 ± 1.85	8.78 ± 1.10	27.6 ± 1.23	13.2 ± 1.16	8.98 ± 0.56	19.23 ± 2.42	10.78 ± 2.10	10.33 ± 0.76
EC (dS m^−1^)	0.25 ± 0.01	0.13 ± 0.02	0.15 ± 0.00	0.24 ± 0.02	0.11 ± 0.00	0.11 ± 0.00	0.24 ± 0.02	0.12 ± 0.01	0.14 ± 0.02
pH	7.54 ± 0.29	8.17 ± 0.10	8.41 ± 0.02	7.35 ± 0.21	8.07 ± 0.14	8.45 ± 0.04	7.70 ± 0.20	8.39 ± 0.12	8.34 ± 0.05
CaCO_3_ (g kg^−1^)	63.5 ± 5.76	42.25 ± 30.39	419.5 ± 33.51	20.75 ± 5.19	45.25 ± 15.9	446 ± 17.26	90 ± 9.63	191.75 ± 35.27	121 ± 57.95
C %	12.09 ± 1.6	5.91 ± 2.26	6.01 ± 0.14	13.27 ± 1.39	5.09 ± 0.58	6.49 ± 0.04	9.77 ± 2.45	3.71 ± 0.13	2.85 ± 0.41
S %	0.01 ± 0.00	0.02 ± 0.01	0.02 ± 0.00	0.03 ± 0.01	0.03 ± 0.00	0.02 ± 0.00	0.01 ± 0.00	0.01 ± 0.00	0.01 ± 0.00
CEC (cmol kg^−1^)	45.38 ± 1.36	34.25 ± 3.30	13.58 ± 1.61	39.68 ± 2.71	35.78 ± 1.31	23.8 ± 0.49	38.65 ± 1.39	23.88 ± 2.22	22.13 ± 1.68
ExCa (cmol kg^−1^)	37.13 ± 1.78	30.5 ± 3.47	12.18 ± 1.68	34.57 ± 3.06	32.95 ± 0.98	22.58 ± 0.4	35.71 ± 1.33	22.46 ± 2.15	19.5 ± 2.22
ExMg (cmol kg^−1^)	7.99 ± 0.52	2.65 ± 1.62	0.57 ± 0.05	3.83 ± 0.71	1.73 ± 0.68	0.48 ± 0.04	1.90 ± 0.36	0.64 ± 0.06	0.83 ± 0.17
ExK (cmol kg^−1^)	0.72 ± 0.02	1.04 ± 0.46	0.84 ± 0.05	1.18 ± 0.32	1.05 ± 0.36	0.64 ± 0.08	0.98 ± 0.12	0.69 ± 0.04	1.4 ± 0.14
ExNa (cmol kg^−1^)	0.08 ± 0.01	0.07 ± 0.02	0.02 ± 0.02	0.1 ± 0.03	0.05 ± 0.01	0.09 ± 0.04	0.07 ± 0.03	0.09 ± 0.01	0.06 ± 0.02
Available P_2_O_5_ (mg 100g^−1^)	2.16 ± 1.15	0.99 ± 0.21	1.73 ± 0.46	2.37 ± 1.10	0.74 ± 0.22	1.24 ± 0.22	0.94 ± 0.29	1.14 ± 0.27	3.77 ± 1.61
N %	0.7 ± 0.07	0.46 ± 0.12	0.18 ± 0.02	0.85 ± 0.13	0.44 ± 0.05	0.21 ± 0.01	0.49 ± 0.05	0.19 ± 0.03	0.19 ± 0.03
SOM %	16.9 ± 1.63	8.45 ± 1.53	2.03 ± 0.13	20.75 ± 0.70	7.13 ± 0.87	2.25 ± 0.13	12.35 ± 2.64	3.53 ± 0.78	2.83 ± 0.34
MB (C, mg kg^−1^)	2419 ± 311	1374 ± 234	534 ± 75	2080 ± 405	838 ± 194	446 ± 49	1407 ± 315	494 ± 80	399 ± 220

**Table 3:** Subgroups obtained in the ANOVAs Post hoc tests (Scheffe) based on soil use ^a^ or soil taxonomy ^b^. Significant differences are considered at confidence level of 95% (alpha < 0.05). Numbers in brackets are the mean of subgroups. Groups are ordered from higher to lower means. Abbreviations as in Table 2.

Parameters	Soil Use ^ab^					Soil Type (FAO) ^b^						
Sand %	Fo-Sh-Cr (25.11)					C-R (32.67)	>	R-M (27.33)	>	M-Hc (24.19)	>	Hc-Hs (19.06)
Silt %	Cr-Sh (37.13)	>	Sh-Fo (34.04)			Hc-Hs-M (39.54)	>	M-R-C (29.55)				
Clay %	Fo-Sh-Cr (39.56)					M-R-Hs-Hc-C (39.56)						
Bulk density (g cm^−3^)	3 (1.22)	>	Sh (0.93)	>	Fo (0.53)	Hc-C (1.15)	>	C-Hs (0.99)	>	R (0.68)	>	M (0.45)
WHC %	Fo (22.89)	>	Sh (12.20)	>	Cr (9.36)	M (24.73)	>	R (19.23)	>	Hs-C (11.73)	>	C-Hc (9.71)
EC (dS m^−1^)	Fo (0.24)	>	Sh-Cr (0.13)			M-R (0.24)	>	C-Hs-Hc (0.13)				
pH	Cr (8.40)	>	Sh (8.21)	>	Fo (7.53)	Hc-C (8.39)	>	C-Hs (8.24)	>	R (7.70)	>	M (7.44)
CaCO_3_ (g kg^−1^)	Cr (328.83)	>	Fo-Sh (75.58)			Hc (432.75)	>	C (156.38)	>	R-M-Hs (52.35)		
C %	Fo (11.71)	>	Sh -3 (5.01)			M (12.68)	>	R (9.77)	>	Hs-Hc (5.87)	>	Hc-C (4.39)
S %	Fo-Sh-Cr (0.02)					M-R-Hs-Hc-C (0.02)						
CEC (cmol kg^−1^)	Fo (41.23)	>	Sh (31.30)	>	Cr (19.83)	M-R (41.23)	>	R-Hs (36.23)	>	C-Hc (20.84)		
ExCa (cmol kg^−1^)	Fo (35.80)	>	Sh (28.64)	>	Cr (18.09)	M-R-Hs (34.17)	>	C-Hc (19.18)				
ExMg (cmol kg^−1^)	Fo (4.57)	>	Sh (1.67)	>	Cr (0.63)	M (5.91)	>	R-Hs-Hc-C (1.26)				
ExK (cmol kg^−1^)	Fo-Sh-Cr (0.95)					M-R-Hs-Hc-C (0.95)						
ExNa (cmol kg^−1^)	Fo-Sh-Cr (0.07)					M-R-Hs-Hc-C (0.07)						
P_2_O_5_ (mg 100g^−1^)	Fo-Cr (2.03)	>	Sh (0.96)			C-M-Hc (2.07)	>	M-Hc-R (1.69)	>	R-Hc-Hs (1.13)		
N %	Fo (0.68)	>	Sh (0.36)	>	Cr (0.19)	M (0.77)	>	R-Hs (0.46)	>	C-Hc (0.19)		
SOM %	Fo (16.66)	>	Sh (6.37)	>	Cr (2.37)	M (18.83)	>	R (12.35)	>	Hs (7.79)	>	C-Hc (2.66)
MB (C mg kg^−1^)	Fo (1968)	>	Sh (902)	>	Cr (460)	M (2250)	>	R-Hs (1206)	>	C-Hc (468)		

(^a^) Fo: Forest; Sh: Shrubland; Cr: Crops. (^b^) M: *mollic Leptosol (C1 and D1)*; R: *rendzic Leptosol (T1)*; Hs: *Leptosol* with shrubland (skeletic C2, and dolomitic D2); Hc: *Leptosol* with crops (skeletic C3, and dolomitic D3); C: *calcaric Regosol (T2 and T3).*

## Data Availability

All data are reported in the figures and tables of the manuscript.

## Supplementary Materials

The following are available online at https://www.mdpi.com/article/10.3390/toxics10010014/s1, Figure S1 Clustering of the A-horizon soil samples using Unweighted Pair Group Method with Arithmetic Mean (UPGMA) cluster analysis with standardized Euclidean distance. (A) Clustering according to both soil properties (physical and chemical variables) and biological and community variables (Shannon Index, Evenness and microbial biomass). (B) Clustering according to soil properties (physical and chemical variables). (C) Clustering according to biological and community variables (Shannon Index, Evenness and microbial biomass). Soils are labelled as parent material (T: Marl and limestone; C: Limestone; D: Dolomites) and soil use (1: Forest; 2: Shrubland; 3: Crops).
