# Supplementary material for: Effects of Soil Quality on the Microbial Community Structure of Poorly Evolved Mediterranean Soils
Source: Toxics. 2022 Jan 3;10(1):14. doi: 10.3390/toxics10010014 (PMC8781153; doi:10.3390/toxics10010014)
Supplement: Supplementary file 1 [file toxics-10-00014-s001.zip › toxics-1418345-supplementary.pdf]

Supplementary Materials: Effects of Soil Quality on the Microbial Community Structure of Poorly Evolved Mediterranean Soils

Antonio Camacho, César Mora, Antonio Picazo, Carlos Rochera, Alba Camacho-Santamans, Daniel Morant, Luis Roca-Pérez, José Joaquín Ramos-Miras, José A. Rodríguez-Martín and Rafael Boluda

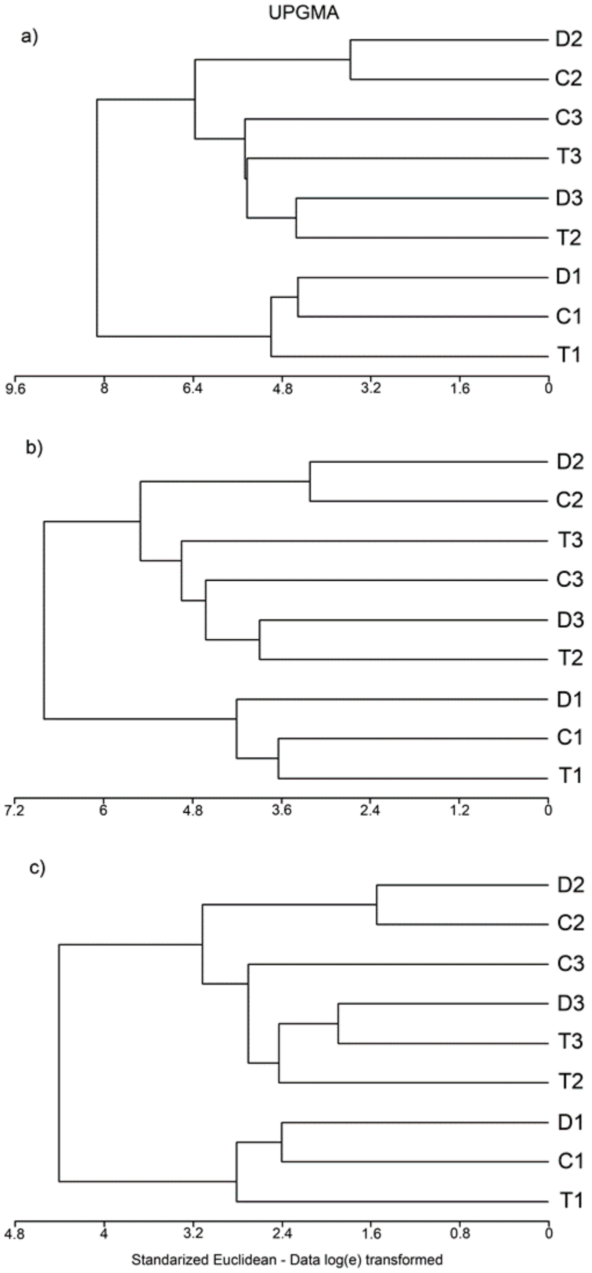

Figure S1. Clustering of the A-horizon soil samples using Unweighted Pair Group Method with Arithmetic Mean (UPGMA) cluster analysis with standardized euclidean distance. (**A**) Clustering according to both soil properties (physical and chemical variables) and biological and community variables (Shannon Index, Evenness and microbial biomass). (**B**) Clustering according to soil properties (physical and chemical variables). (**C**) Clustering according to biological and community variables (Shannon Index, Evenness and microbial biomass). Soils are labelled as parent material (T: Marl and limestone; C: Limestone; D: Dolomites) and soil use (1: Forest; 2: Shrubland; 3: Crops).
